# Negatively Charged In-Plane and Out-Of-Plane Domain Walls with Oxygen-Vacancy Agglomerations in a Ca-Doped Bismuth-Ferrite Thin Film

**DOI:** 10.1021/acsaelm.1c00638

**Published:** 2021-09-24

**Authors:** Ulrich Haselmann, Y. Eren Suyolcu, Ping-Chun Wu, Yurii P. Ivanov, Daniel Knez, Peter A. van Aken, Ying-Hao Chu, Zaoli Zhang

**Affiliations:** † Erich Schmid Institute of Materials Science, Austrian Academy of Sciences, Leoben 8700, Austria; ‡ Department of Materials Science and Engineering, Cornell University, Ithaca, New York 14850, United States; § Max Planck Institute for Solid State Research, 70569 Stuttgart, Germany; ∥ Department of Materials Science and Engineering, National Chiao Tung University, Hsinchu 30010, Taiwan; ⊥ Department of Materials Science & Metallurgy, University of Cambridge, Cambridge CB3 0FS, U.K.; # School of Natural Sciences, Far Eastern Federal University, Vladivostok 690950, Russia; ¶ Graz Centre for Electron Microscopy, Austrian Cooperative Research, Graz 8010, Austria; ∇ Institute of Material Physics, Montanuniversität Leoben, Leoben 8700, Austria

**Keywords:** BiFeO_3_, oxygen vacancy, ordering in oxygen vacancy plates, charged domain wall, aberration-corrected STEM, domain-wall pinning, domain-wall nanoelectronics

## Abstract

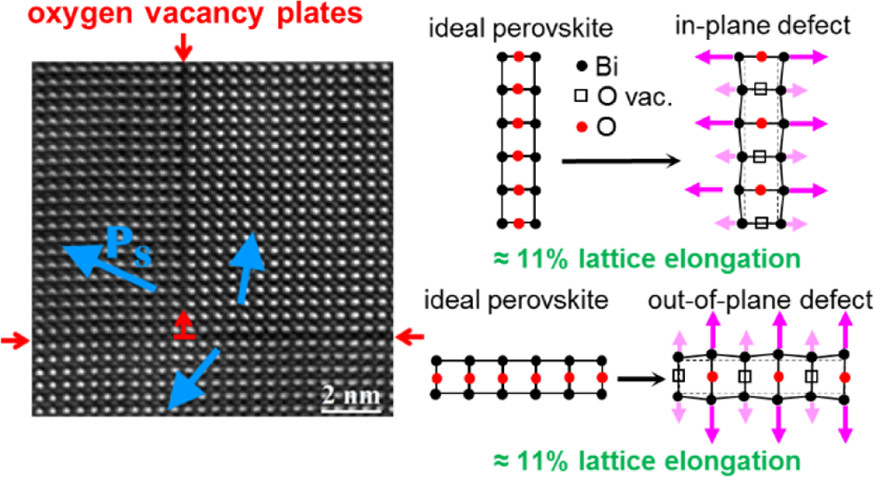

The interaction of oxygen vacancies and ferroelectric domain walls is of great scientific interest because it leads to different domain-structure behaviors. Here, we use high-resolution scanning transmission electron microscopy to study the ferroelectric domain structure and oxygen-vacancy ordering in a compressively strained Bi_0.9_Ca_0.1_FeO_3−δ_ thin film. It was found that atomic plates, in which agglomerated oxygen vacancies are ordered, appear without any periodicity between the plates in out-of-plane and in-plane orientation. The oxygen non-stoichiometry with δ ≈ 1 in FeO_2−δ_ planes is identical in both orientations and shows no preference. Within the plates, the oxygen vacancies form 1D channels in a pseudocubic [010] direction with a high number of vacancies that alternate with oxygen columns with few vacancies. These plates of oxygen vacancies always coincide with charged domain walls in a tail-to-tail configuration. Defects such as ordered oxygen vacancies are thereby known to lead to a pinning effect of the ferroelectric domain walls (causing application-critical aspects, such as fatigue mechanisms and countering of retention failure) and to have a critical influence on the domain-wall conductivity. Thus, intentional oxygen vacancy defect engineering could be useful for the design of multiferroic devices with advanced functionality.

## 1 Introduction

Multiferroic materials showing a coupling between ferroelectric and magnetic order parameters are referred to as magnetoelectric multiferroics and have attracted much attention in the past years.^1−4^ For example, in a magnetoelectric multiferroic, an electrical stimulation results in an effect on the magnetic order parameters.^5−7^ This behavior is intriguing for numerous applications such as new kinds of memory devices promising significantly improved speed and storage density,^8,9^ spin valves, spintronic devices, and sensors.^10^ Single-phase multiferroic materials with high Néel and Curie temperatures, which are critical for practical applicability, are relatively rare. One of those is BiFeO_3_ with a high antiferromagnetic Néel temperature of *T*_N_ ≈ 370 °C and an even higher ferroelectric Curie temperature of *T*_C_ ≈ 830 °C. In particular, the control of the antiferromagnetic domains *via* the manipulation of the ferroelectric ones has been successfully demonstrated.^11^ Additionally, the domain walls of BiFeO_3_ have a higher conductivity than the domains itself,^12^ promising the prospect of resistance-switching devices in the field of domain-wall nanoelectronics.^13^

Perovskite oxides such as BiFeO_3_ become even more versatile through the incorporation of substitutional elements on the A (Bi) or B-site (Fe), which enables us to manipulate and design the electronic and magnetic properties within certain limitations.^14,15^ Ca as an A-site dopant has been shown to tune the material’s behavior from antiferromagnetic toward ferromagnetic^16^ but also to enhance the magnetoelectric coupling^17^ and to enable a conductivity modulation through the application of an electric field.^18^ Ca doping in BiFeO_3_ can induce oxygen vacancies because the replacement of Bi^3+^ with Ca, which is an alkaline earth metal and therefore cannot have a higher oxidation state than 2+, has a hole doping effect. Stoichiometrically Ca doping in Bi_1–*x*_Ca_*x*_FeO_3−δ_ would lead to δ = *x*/2 oxygen vacancies.^18−20^ It has been reported that for doping ratios of *x* ≥ 0.2, the oxygen vacancies arrange in ordered superstructures.^18,20−23^ Thereby, Ca is not uniformly distributed but can segregate to the energetically most favorable position.^19,24,25^ When relatively thick films are under a compressive (tensile) strain, these superstructures are expected to be arranged parallel (perpendicular) to the interface, as shown in a study for LaCoO_3–*x*_ films.^26^ For thin, tensile-strained films where the surface energy becomes dominant compared to the bulk energy, the effect is seen, in order to minimize the surface energy, the perpendicular arranged oxygen defects become parallel.^27^ While for doping ratios *x* ≤ 0.1, there are reports of some vacancy ordering under certain conditions,^28^ other results indicate that they are in most cases not ordered in superstructures.^20,21,28^

Improved magnetic behavior^16^ and the coupling between the electric and magnetic ordering parameters^17^ alone are often not enough for potential device applications because regions where the ferroelectric polarization has been switched are at times unstable and do not stay in their switched state, but relax back to the original state. This process is known as “retention failure” and can lead to a loss of functionality of the device.^29^ Generally, it has been accepted that electrostatic boundary conditions at the ferroelectric/electrode interface are a cause for the back switching. Strong fields that lead to depolarization can arise at uncompensated interfaces^29−31^ as well as impurity defects.^29^ However, defects are also a method to counter retention failure by acting as pinning centers hindering the movement of domain walls but simultaneously leading to the fatigue phenomena by degradation of switchable polarization.^29,32^ Besides phase boundaries,^33^ dislocations,^34^ and pre-existing ferroelastic domains,^35^ these defects can also be oxygen vacancies,^36−38^ leading to another potential improvement of material properties by Ca doping.

In this study, we investigate a Ca-doped Bi_0.9_Ca_0.1_FeO_3−δ_ (BCFO) film deposited on a SrTiO_3_ substrate covered with a 55 nm thick SrRuO_3_ interlayer. While there are no ordered superstructures, there are randomly ordered features in the atomic resolution image, which are either parallel or perpendicular to the interface. We show by atomic-resolution scanning transmission electron microscopy (STEM) with high-angle annular dark-field (HAADF), electron energy-loss spectroscopy (EELS), and the comparison with characteristic results from previous works that these features are oxygen-deficient planar defects and, simultaneously, negatively charged domain walls with a tail-to-tail configuration. The atomic-resolution experimental results are compared with STEM image simulations, and the HAADF images are evaluated with software scripts to determine the atomic-column locations, intensities, interatomic spacings, and ferroelectric polarization. Thereby, we found that while reduced intensities on the Fe sites of the planar defects do not indicate any Fe vacancies, either an increased Ca concentration or Bi-vacancy concentration is detected on the A-sites around the defect. The film is under a compressive strain, which should favor planar defects parallel to the interface, but the defects occur in both directions, showing the same characteristics and presumably containing a similar amount of oxygen vacancies. Our work confirms that charged domain walls in BiFeO_3_ oriented both in-plane and out-of-plane can be coupled to a local accumulation of oxygen vacancies. This coupling is expected to lead to pinning of the domain walls and an improved resistivity against retention failure.

## 2 Experimental Details

### 2.1 Thin-Film Fabrication

The thin film was fabricated by pulsed laser deposition (PLD) using a KrF excimer laser (Coherent, Inc., 248 nm) with a pulse repetition rate of 10 Hz on a single crystalline (001)-oriented SrTiO_3_ (STO) (Shinkosha, Co., Ltd.) substrate. Before the thin-film deposition, the substrate was cleaned by acetone and ethanol in an ultrasonic cleaner. First, using a total deposition time of 30 min, a 55 nm thick SrRuO_3_ (SRO) intermediate layer was epitaxially grown with an energy of 220 mJ/pulse onto the STO substrate. Afterward, a 10% Ca-doped Bi_0.9_Ca_0.1_FeO_3−δ_ (BCFO) thin film was deposited with a deposition time of 45 min and an energy of 220 mJ/pulse onto the SrRuO_3_ intermediate layer, resulting in a film thickness of 60 nm. The vacuum chamber was first kept at a pressure of about 10^–7^ Torr. During the deposition, the substrate was maintained at a temperature of 700 °C with an oxygen environment of 100 mTorr. Cross-sectional samples for the TEM investigations were prepared using a Helios Nanolab FIB-SEM by a standard focused ion beam protocol.^39−41^

### 2.2 Data Acquisition

A probe aberration-corrected (DCOR, CEOS GmbH) JEOL JEM-ARM200F with a cold field-emission electron source operated at 200 kV and equipped with a Gatan GIF Quantum ERS spectrometer was used for all STEM analyses. STEM imaging and EELS analysis were performed at a semi convergence angle of 20 mrad, resulting in a probe size of 0.8 Å.^42^ Detailed imaging parameters for the HAADF images can be found in Table S1. For the EELS spectral image, a collection semi-angle of 111 mrad, a pixel time of 10 ms with 48 * 170 pixel, and an energy channel width of 1 eV have been used. High-resolution transmission electron microscopy (HRTEM) using a JEOL JEM 2100 F with an image-side C_S_ corrector, an acceleration voltage of 200 kV, and a Gatan Orius SC1000 camera was additionally used to evaluate the strain state of the film.

### 2.3 Data Evaluation

The strain analysis was performed with the Geometric Phase Analysis (GPA) software package v4.0 from HREM research company for Digital Micrograph 2.3 from Gatan. For the analysis of HAADF images concerning intensities, site spacings, and ferroelectric polarization, two homemade MatLab scripts written in MathWorks Inc. MatLab (version R2017b) were used. Prior to applying the scripts, the experimental images were filtered with a principal component analysis (PCA) script for Digital Micrograph written by S. Lichtert, T. Hayian, and J. Verbeeck to reduce image noise.^43^ At the beginning of the first MatLab scrip, the locations of atomic columns in the image are ascertained by searching for local maxima. Gaussian fitting with sub-pixel precision is used to refine the peak locations and then as a basis for Voronoi tessellation. These received cells are utilized to determine the absolute intensity from the unfiltered image by integrating over a defined area corresponding to each atomic column. Columns at the edge of the image that lie not entirely in it are excluded.^24,44^ The integrated column intensity is a robust measurement, as it is insensitive to the probe shape and the defocus.^24,45^ The second MatLab script was written to evaluate (i) the fitted atomic-column positions from the first script according to the spacings between the atomic sites, (ii) the polarization due to shifts of the central Fe atom, and (iii) to visualize the data. The EELS spectra were processed using the built-in functions of digital micrograph and additionally filtered in OriginPro 2016 using a Savitzky–Golay filter with a window of nine data points for denoising. HAADF-image simulations with the software Dr. Probe^46^ were performed for a sample thickness of 30 nm (76 BiFeO_3_ unit cells), an acceleration voltage of 200 kV, a semi convergence angle of 20.9 mrad, a spherical aberration of 0 μm, a defocus value of 0 nm, an effective source radius of 0.10 Å, a scanning step size of 10 pm, and a HAADF detector ranging from 49 to 250 mrad. Those parameters were chosen so that the simulation results are comparable with the experimental parameters used for HAADF acquisition.

## 3 Results

### 3.1 Large-Scale Structure of the Film

STEM investigations of the overall thin film cross-section showed that the SRO as well as the BCFO film have grown epitaxially on the STO substrate, as can be seen in Figure S1. The in-plane strain is only slightly increased over the thickness of the 55 nm wide SRO layer and the first 20 nm of the film covered in that image frame, while the out-of-plane strain is immediately increased at the STO/SRO interface and only marginally decreases over the width of the SRO thin film layer and the adjacent BCFO thin film. These strain characteristics are expected for a good quality epitaxial growth because the pseudocubic SRO and pure BiFeO_3_ (BFO) lattice parameters are approximately 0.6^47^ and 1.5%^10,48^ larger than the lattice parameter for bulk STO. Therefore, the thin films are compressed in the in-plane direction and enlarged in the out-of-plane direction. For the BCFO film, the lattice parameters received from the strain maps in Figure S1b,c are 3.93 ± 0.01 Å for the in-plane direction and 4.01 ± 0.01 Å for the out-of-plane direction, resulting in an enlargement in the out-of-plane direction by 2.0 ± 0.5%. Determining the lattice parameter with HRTEM measurements, which have the advantage that drift distortions are minimized compared to STEM, 3.91 ± 0.01 Å for the in-plane lattice parameter and 4.02 ± 0.01 Å for the out-of-plane lattice parameter are received, resulting in measured enlargement of the out-of-plane direction of 2.9 ± 0.5%.

**Figure 1:**
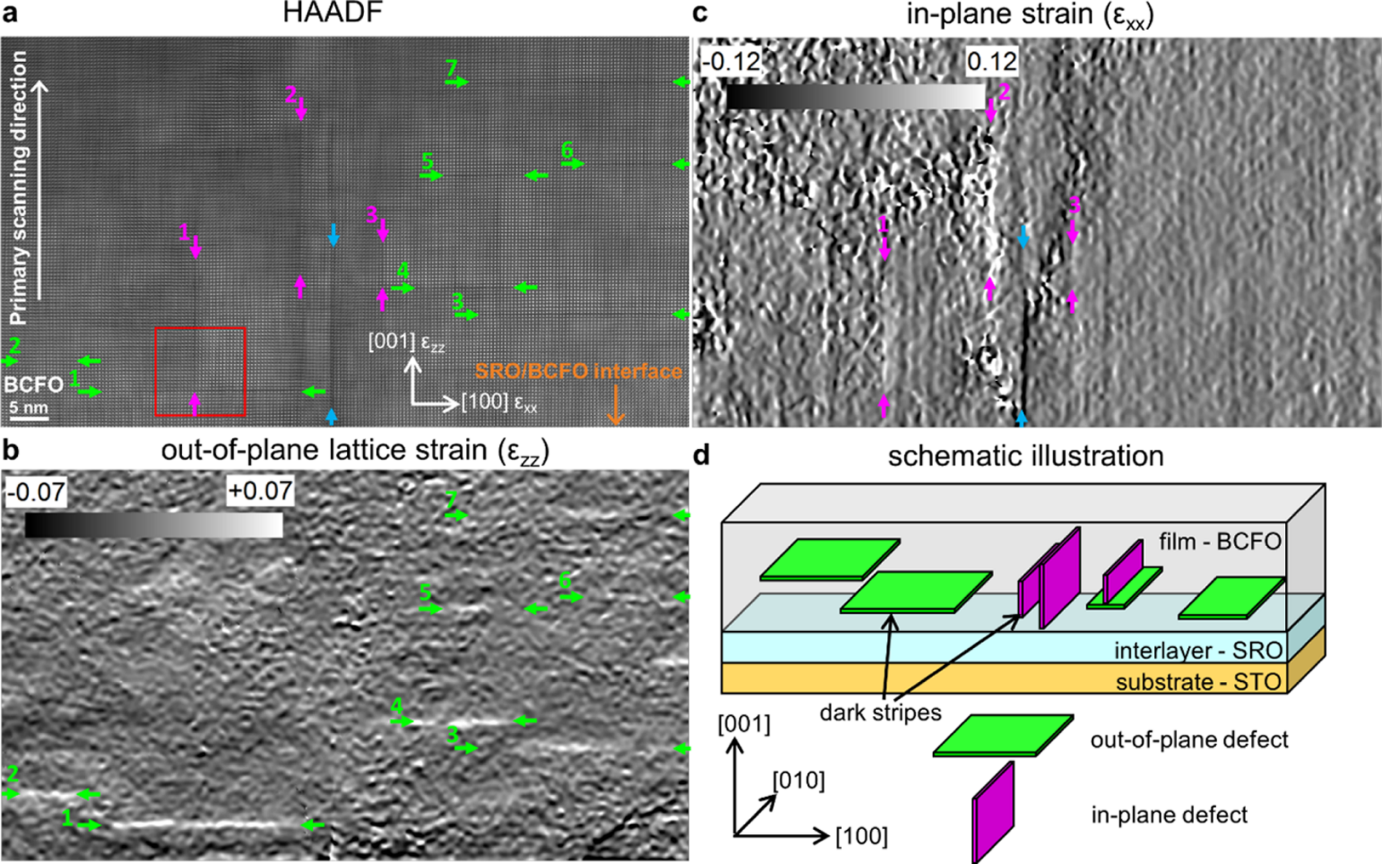
Large-scale structural mapping of the BCFO thin film and its defects. (a) HAADF image of the BCFO film along the [010]_pc_ zone axis. The red square indicates the area shown and analyzed in Figure 2. (b) Map of the out-of-plane strain (ε_*zz*_) and (c) map of the in-plane strain (ε_*xx*_). The colored arrows mark the beginning and the end of the dark stripes found in the HAADF image. Green arrows mark dark stripes, where ε_*zz*_ is increased, and are therefore called out-of-plane defects, while magenta arrows indicate dark stripes, where ε_*xx*_ is increased, and are therefore called in-plane defects. The blue arrows indicate an edge dislocation relaxing the in-plane strain, which starts at the SRO/BCFO interface (not shown here). (d) Schematic illustration of the thin film and the two types of defects (the best contrast can be seen in the digital version).

Figure 1a shows a large-scale (low magnification) HAADF image of the BCFO film covering almost the entire epitaxial layer. The SRO/BCFO interface is on the bottom side of the imaging frame and not visible. Noticeable in the HAADF image are several stripes with darker contrast than the surrounding areas, which are either parallel to the [100]_pc_ (subscript pc refers to pseudo-cubic) or the [001]_pc_ direction. Their starting and endpoints are marked with colored arrows, and they are numbered. The map of the out-of-plane lattice strain in Figure 1b shows that the dark lines parallel to the [100]_pc_ (in-plane) direction, which are marked with green arrows and numbers, coincide with an enlarged strain and therefore with an enlarged lattice parameter in the out-of-plane direction. Because the out-of-plane lattice parameter is enlarged, they are called *out-of-plane defects*. Similarly, in the in-plane strain map shown in Figure 1c, dark lines parallel to the [001]_pc_ (out-of-plane) direction are marked by the mangenta colored arrows, and are called *in-plane defects*, because of their enlarged lattice parameter in the in-plane direction. Figure 1d shows a schematic illustration of the film system and the in-plane and out-of-plane defects. The blue arrows in Figure 1a,c mark a single misfit/edge dislocation, which originates at the SRO/BCFO interface and locally reduces the compressive strain. These dislocations are not very frequent and occur approximately once per 500 nm interface length and therefore will not cause a significant relaxation of the film. These edge dislocations are not the focus of this study because they have already been investigated extensively for BiFeO_3_ thin films under compressive strain.^49,50^

### 3.2 Analysis of In- and Out-Of-Plane Defect with a Contact Point

**Figure 2:**
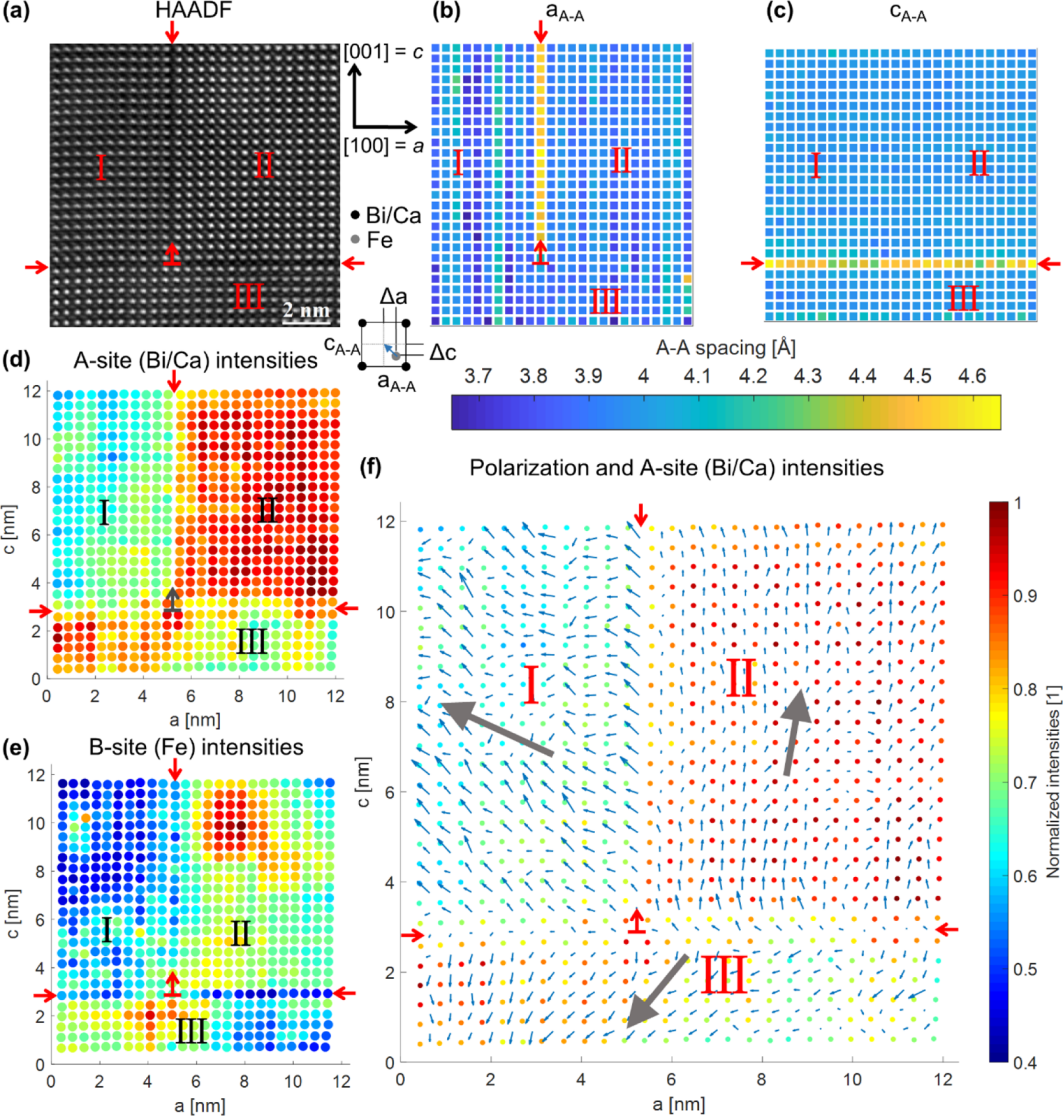
Analysis of high-resolution STEM data of an in-plane and out-of-plane defect, which have a common point of contact (region indicated by the red square in Figure 1). (a) HAADF image showing two defects marked with red arrows. The defects separate the image into three areas designated with roman numbers I to III. On the top right side of the HAADF image, the [100]_pc_ and [001]_pc_ axes are indicated as *a* and *c*. At the bottom of the right side is a schematic illustration of the electrical polarization due to shift of the Fe atom from the center of the pseudo-cubic cell. (b) Map of interatomic distances of the A-sites (Bi and Ca atoms) in the a (in-plane) direction. The in-plane defect marked by the red arrows can be clearly seen by the enlarged lattice parameter. (c) Map of interatomic A-site distances in the *c* (out-of-plane) direction. The out-of-plane defect can be clearly observed by the enlarged lattice parameter. (d) A-site intensities (Bi and Ca positions) in the HAADF image normalized to the maximal A-site intensity. (e) B-site (Fe) intensities in the HAADF image normalized to the maximal B-site intensity. (f) Map of the electrical polarization due to the shift of the Fe atom overlayed on the fitted positions and intensities of the A-site atoms. The gray arrows in the regions I–III indicate the average polarization direction and magnitude relative to each other. A version optimized for colorblind readers can be found in Figure S2.

In Figure 1a, the red square indicates an area, where one in-plane defect and one out-of-plane defect (for the difference between the two and the definitions, see Figure 1d) touch each other. A more detailed analysis can be seen in Figure 2. In the HAADF image shown in Figure 2a, the in-plane and out-of-plane defects are indicated by red arrows. These defects divide the image in three areas, which are numbered from I to III. The pseudo-cubic [100] direction is defined as the axis *a*, while the [001] direction is defined as *c*. Figure 2b shows the map of interatomic distances between A-sites (a_A–A_) in the *a* (in-plane) direction. It can be clearly seen that along the *a* direction, there is an elongation of interatomic A-site distances at the in-plane defect, whose position is indicated again by red arrows and ends at the touching point with the out-of-plane defect (marked by the arrow with a crossbar at the beginning). The out-of-plane defect shows no lattice enlargement in the *a* direction and is completely invisible in the map. The same interatomic distances in the *c* (out-of-plane) direction (c_A–A_) can be found in Figure 2c. In contrast to Figure 2b, the out-of-plane defect can now be observed by an enlarged A–A spacing, while the in-plane defect is not visible. The map of the normalized A-site intensities, where Bi and Ca atoms are sitting, is presented in Figure 2d. In region II, the A-site intensities are the highest, while in region I, they are reduced by approximately 20%. The reason for that is that the images of the A-sites look different in region I and are slightly elongated compared to region II. The root cause for that is probably, that due to the out-of-plane defect, region I is not exactly aligned in zone-axis orientation and is slightly tilted. In region III, the A-site intensities change in a continuous way from the left to the right side of the image. In Figure 2e, the normalized B-site intensities (Fe atoms) are displayed. It is quite noticeable that especially at the in-plane defect but also at the out-of-plane defect, the intensities of the B-site positions are reduced. In Figure 2f, a map of the electric polarization^51,52^ is displayed, which is obtained from the shift of the Fe atom away from the center of the pseudo-cubic cells, whose edges are formed by the Bi atoms sitting at the corner of the surrounding A-sites (for a schematic illustration see Figure 2a on the right bottom side). The projection of the polarization direction is thereby the vector sum of the negative shift of the Fe atom −Δ*a* in the *a* direction and −Δ*c* in the *c* direction.^19,32,53−55^ The color bar on the right side of Figure 2f is also valid for Figure 2d,e. It is clear from this map that each of the three regions has a prevalent polarization orientation excluding some minor deviations. The big gray arrows indicate the relative size and orientation obtained from averaging all polarization vectors in each region. In bulk BiFeO_3_, the ferroelectric polarizations are oriented along the pseudocubic [111] orientation. However, in thin films, especially in the proximity of defects, it has already been reported before that the polarization can differ from this direction.^19,49^ The defects clearly represent charged domain walls (CDWs) between all three regions in a tail-to-tail configuration. Between regions I and II, they are either of the kind 71 or 109°, assuming that the polarizations are indeed oriented along the [111] direction and the deviations from it are in fact measurement errors. Because the b direction ([010]_pc_) can only be observed in projection, we cannot differentiate between 71 and 109° CDWs. Region III deviates from the [111] direction as well. Between regions I and III, there is also a 71 or 109° CDW, and between II and III, there is a 109 or 180° CDW. An optimized version for colorblind readers of Figure 2 is displayed in Figure S2.

### 3.3 Analysis of In-Plane Defects

**Figure 3:**
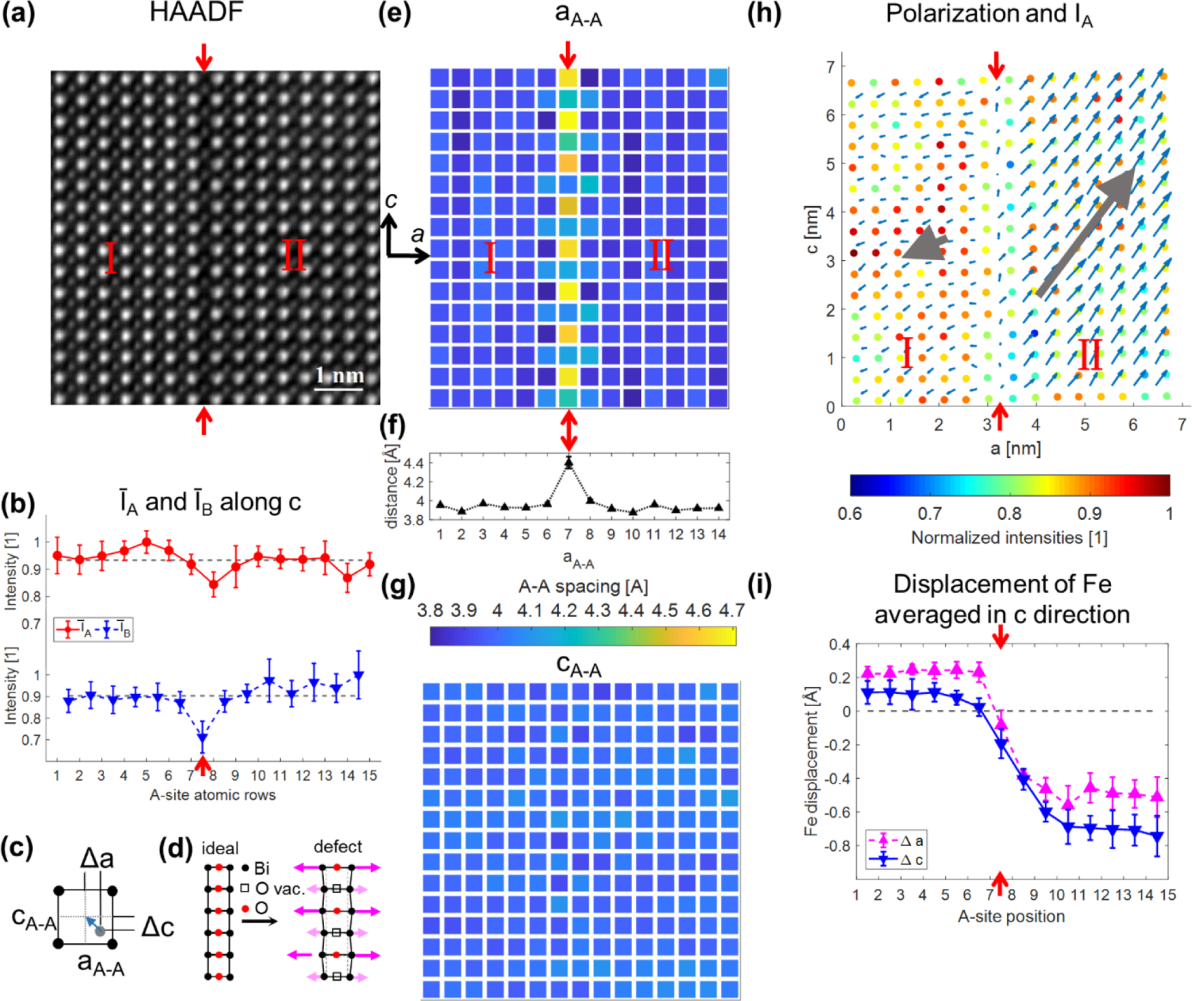
Exemplary analysis of an in-plane defect. (a) HAADF image of the in-plane defect marked by red arrows. (b) A- and B-site intensities (*I̅*_A_ and *I̅*_B_) averaged along the *c* direction. The A-sites are numbered in integers, while the B-sites are counted as integers of ±0.5 (half sites). (c) Schematic illustration of the electric polarization due to shift of the central Fe atom by Δ*a* and Δ*c* in the pseudo-cubic perovskite unit cell. (d) Illustration of the arrangement of the A-site atoms in the ideal perovskite and at the defects including the oxygen stoichiometry in the FeO_2−δ_ plane (see Discussion section).^19^ (e) Map of interatomic A–A-site distances in the a direction (*a*_A–A_). The defect is marked by red arrows and shows an enlarged lattice parameter with an alternating pattern of one site having a larger elongation, whereas the next one has a less pronounced one. The color bar can be found in (g). (f) A–A-site distances averaged along the *c* direction showing the average elongation at the defect. (g) Map of interatomic A–A-site distances in the c direction (*c*_A–A_). No variation along the defect is visible. (h) Map of the electric polarization due to shift of Fe atoms overlayed on the fitted positions and intensities of the A-site atoms (*I*_A_). (i) Displacement of Fe atoms separated in Δ*a* and Δ*c* components averaged in the *c* direction. A version optimized for colorblind readers can be found in Figure S3a.

To explore the defects in more detail, an in-plane defect (for the definition see Figure 1d) is being analyzed separately in this section. Figure 3a shows the HAADF image of a different in-plane defect (than the one showed in Figure 2). The red arrows mark the defect, which separates the imaged area in regions I and II. In Figure 3b, the HAADF intensities normalized to the maximum A- or B-site intensities, which have been averaged along the *c* direction, are shown. The gray dashed lines indicate the average of the measured intensities. The B-site intensities (*I̅*_B_) are reduced exactly at the defect. Similarly, the A-site intensities (*I̅*_A_) in region II on the right side of the defect are noticeably reduced (atomic row 8), while on the left side of the defect (region I), there is a less pronounced intensity decrease on atomic row 7. Figure 3e shows the interatomic distances between A-sites (*a*_A–A_) in the *a* direction (in plane; the associated color bar can be found in Figure 3g). These interatomic distances clearly show a significant elongation exactly at the defect compared to the ideal perovskite structure, as can be seen by the lattice spacing averaged in the *c* direction in Figure 3f. The distortion is not uniform along the defect, but shows an alternating pattern, where one A–A atomic distance shows a larger elongation and the subsequent one has a less pronounced elongation. The analysis of the mean lattice elongation at in-plane defects (five defects analyzed including the in-plane defect shown in Figure 2) yields 11.4 ± 0.3% of the in-plane lattice parameter. The difference between the values for the lattice elongation of the in-plane defects shown in Figures 2 and 3 is within the measurement errors. The small statistic variance indicates that all the defects show essentially the same elongation. A summary of the lattice parameters and the lattice elongation values can be found in Table 1. A schematic illustration of this observed defect compared to the ideal perovskite structure can be seen in Figure 3d. In comparison, the interatomic A-site distances in the *c* direction show no influence of the defect (Figure 3g). The map of the electric polarization due to the shift of Fe atoms from the center of A-site quartets (schematically depicted in Figure 3c) is presented in Figure 3h. From this polarization map, it is obvious that the defect also marks a tail-to-tail CDW between regions I and II. The big gray arrows in Figure 3h show the averaged polarization direction and magnitude for regions I and II. The magnitude of the polarization vector in region II is larger than in region I, which is also depicted by the correct ratio of the norms of the gray vectors in Figure 3h. The reason for that can be seen in the A sites (Bi) in region II (Figure 3a), which are not nicely circular like the A-sites in region I, but elongated toward the northeast direction of the image. This either seems to stem from a slight tilt introduced by the defect or there is an astigmatism in region II, while there is none in region I. This slightly increases the magnitude of the polarization in region II, but it does not influence the general polarization direction or the relative polarization relationship between regions I and II. From these directions, we can deduce that it can either be a 109° CDW or a 180° CDW. Figure 3i plots the shift of the Fe atoms from the center separated in the Δ*a* and Δ*c* components averaged along *c* direction, which correspond to the deviation of the Fe atom from the center of the four surrounding A-sites in *a* and *c* directions, as schematically depicted in Figure 3c. The Fe displacement values shift exactly at the defect.

**Table 1:** Lattice Parameters of the BCFO Thin Film and Average Elongation of the In-Plane and Out-Of-Plane Defects With the Average Scanning Step Size Used for the Defect Analysis

		defects		
	lattice parameter [Å]	elongation [%]	elongation [Å]	average step size [pm]
**in-plane**	3.93 ± 0.01	11.4 ± 0.3	0.45 ± 0.01	7.46
**out-of-plane**	4.01 ± 0.01	10.7 ± 1.0	0.43 ± 0.04	12.48

### 3.4 Analysis of Out-Of-Plane Defects

**Figure 4:**
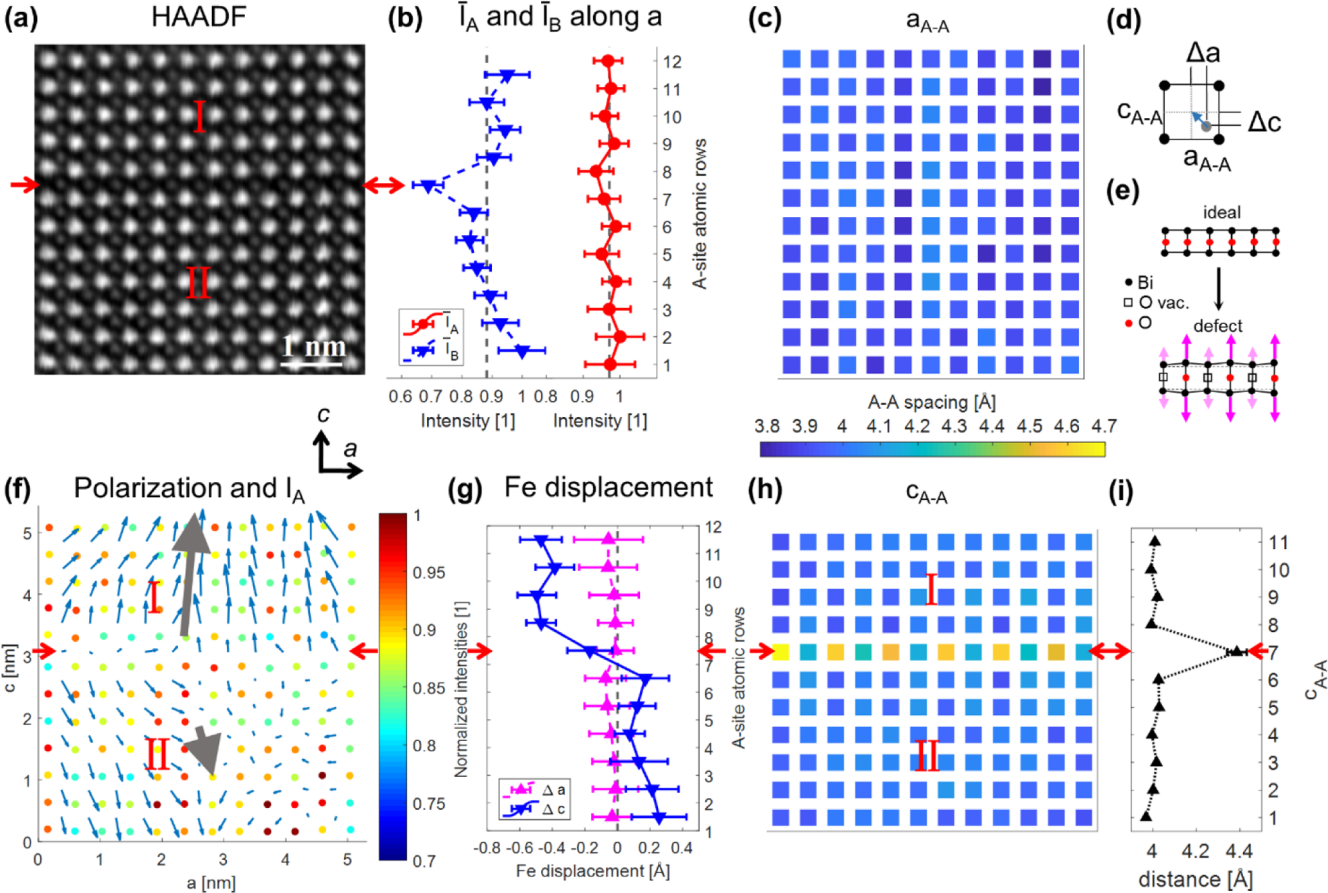
Exemplary analysis of an out-of-plane defect. (a) HAADF image of the defect marked with red arrows. (b) A- and B-site intensities (*I̅*_A_ and *I̅*_B_) averaged along the *a* direction. The A sites are numbered in integers, while the B sites are counted as integers of ±0.5 (half-sites). (c) Map of interatomic A-site distances in the *a* direction (*a*_A–A_). No variation along the defect is visible. (d) Schematic illustration of the electric polarization due to shift of the central Fe atom by Δ*a* and Δ*c* in the pseudo-cubic perovskite unit cell. (e) Schematic presentation of the A-site atom arrangement in the ideal perovskite and at the defects including the oxygen stoichiometry in the central FeO_2−δ_ plane of the defect (see Discussion section).^19^ (f) Map of the electric polarization due to the shift of the Fe atom overlayed on the map of the fitted positions and intensities of the A-site atoms (*I*_A_). A version optimized for colorblind readers can be found in Figure S3b. (g) Displacement of Fe atoms displayed for Δ*a* and Δ*c* averaged in the a direction. (h) Map of interatomic A-site distances in the c direction (*c*_A–A_). The defect is marked by red arrows and shows an enlarged lattice parameter with a typical alternating A–A distances. (i) A–A-site distances averaged along the c direction displaying an average lattice elongation at the defect.

In this section, an exemplary analysis of an out-of-plane defect (for the definition, see Figure 1d) is presented and compared to the previously described in-plane defects. In Figure 4a, an HAADF image of a defect, which separates the imaged area in regions I and II, is presented. The red arrows mark the position of the defect. The intensities for A- and B sites averaged along the *a* direction are shown in Figure 4b. The averaged intensities of the B sites (*I̅*_B_) show, similar to the in-plane defect in Figure 3b, a significant intensity decrease at the defect location. The A-site intensities display a weak intensity decrease in atomic row 8 above the defect, but no significant intensity difference can be seen in atomic row 7 below the defect. The map of the interatomic distances between A-sites in the *a* direction (*a*_A–A_) in Figure 4c is opposite to the same map for the in-plane defect in Figure 3e; there are no local distortions because the present defect is an out-of-plane defect. Instead, the map of the interatomic distances in the *c* direction (*c*_A–A_) in Figure 4h shows the same alternating lattice-elongation pattern as for the in-plane defect, with one A–A couple having a large elongation and the subsequent one having a less pronounced elongation. The lattice spacing averaged in the *a* direction is displayed in Figure 4i, showing a significant overall increase at this defect. The analysis of the mean lattice elongation at out-of-plane defects (5 defects analyzed including the out-of-plane defect of Figure 2) shows a lattice parameter elongation of 10.7 ± 1.0%. The difference between the values for the lattice elongation of the out-of-plane defects in Figures 2 and 4 is also within the measurement errors. No large difference in the lattice elongation of the out-of-plane defects compared to the in-plane defects could be observed, indicating that the nature of all defects is the same. The summary of the out-of-plane lattice-elongation values can be found in Table 1. The map of the electric polarization is presented in Figure 4f. Again, the defect turns out to be a tail-to-tail CDW. The gray arrows show the averaged polarization moment for the regions I and II, displaying, besides the direction, also the relative magnitude of the polarization in regions I and II. The absolute value of the polarization vector in region I is larger than in region 2. Figure 4g shows the Fe site shift averaged in the *a* direction and demonstrates that the orientation of the Fe displacements shifts exactly at the defect as observed for the in-plane defects.

### 3.5 Atomic Resolution EELS at the Defects

**Figure 5:**
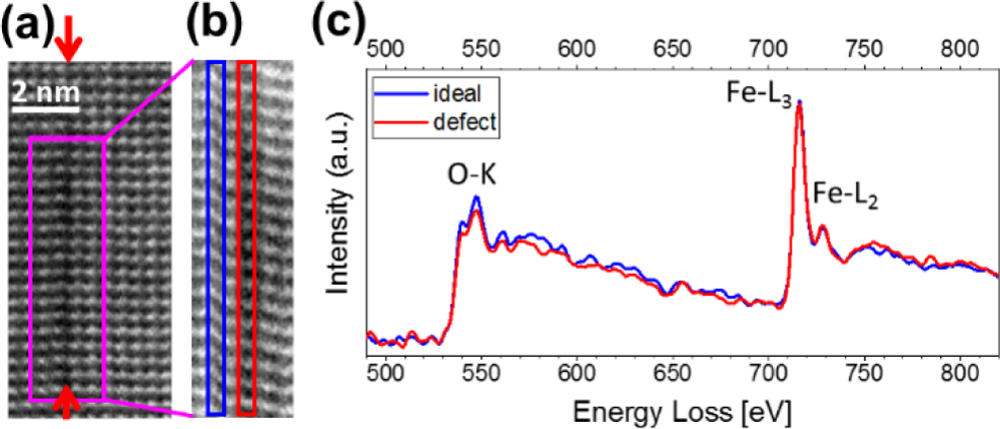
Atomic resolution EELS study at the defects. (a) HAADF image of the defect. The magenta rectangle indicates the area where the (b) spectrum image was recorded. The red rectangle marks the area of the defect and the blue one the undistorted perovskite area for comparison. (c) EELS spectra recorded on the defect (red) and beside the defect (blue).

After revealing information on the structural characteristics, analytical investigations on the defects were performed using STEM_EELS. In Figure 5a, the HAADF image of an in-plane defect is shown. The area of interest for the acquired EELS core-loss spectra is highlighted with a magenta rectangle. The spectral signals of a width of approximately one pseudo-cubic unit cell along the defect (red rectangle in the EELS map in Figure 5b) and of the undistorted perovskite (blue rectangle) are compared. The distance between the O–K and Fe–L_3_ onsets for on and off the defect remains the same, which indicates that the Fe-oxidation state remains the usual Fe^3+^ in the area of the defect.^56^ Additionally, this is supported by the fact that there is no change in the ELNES fine structure of the Fe or the O edge.^57^ Thus, the Fe-oxidation state is not changed by the defect. Concerning the intensity of the O–K edge, the signal from the defect (red curve in Figure 5c) seems slightly weaker than the signal from the undistorted perovskite, indicating that the defect may indeed contain less oxygen (oxygen vacancies).

## 4 Discussion

**Figure 6:**
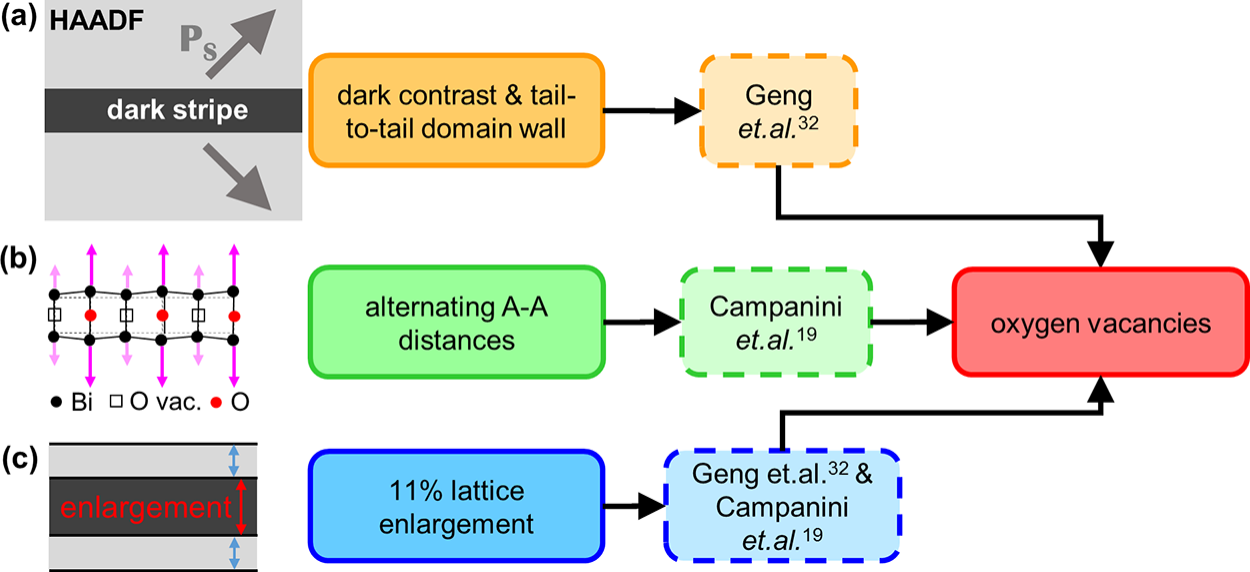
Schematic illustration of the three indicators for the reason of the dark stripes being the agglomeration of oxygen vacancies: (a) Tail-to-tail CDW (negatively charged) with dark contrast in the HAADF images. The accumulation of oxygen vacancies (positively charged) would compensate for the CDW.^32^ (b) A–A distances across the dark stripe show one larger elongation, followed by one smaller elongation, which is linked to a special ordering of oxygen vacancies reported by Campanini *et al.*(19) (c) Average lattice expansion of around 11% was measured. The same was reported by Geng *et al.* for δ = 1 referring to the FeO_2−δ_ plane.^32^ That means every second oxygen should be missing there, which again fits to the oxygen vacancies and structural changes reported by Campanini *et al.*(19)

Considering the data gathered about the dark stipes, their exact nature becomes evident. Because the areas adjacent to the dark stripes are slightly tilted relative to each other (*e.g.,*Figure 2 region I and II and Figure 3), annular bright field (ABF) images could not be used to analyze the reason for the dark stripes and other indications had to be used. An overview of the three main indicators discussed in the following in detail can be found in Figure 6. In the literature, dark stripes in HAADF images of perovskites have been described as structural modifications due to the local accumulation of oxygen vacancies.^19,20,26,32,58^ Geng *et al.* were able to show directly with EELS for an undoped BFO film that the dark stripes are indeed oxygen-deficient. They could confirm this by etching combined with XPS and the fact that films grown with less oxygen partial pressure showed longer dark stripes in the cross-sectional samples.^32^ Geng *et al.* also observed that the oxygen-vacancy stripes always resulted in a CDW with a tail-to-tail configuration^32^ (see Figure 6a), where the negative charge of the tail-to-tail domain walls compensates for the positively charged oxygen vacancies.^59,60^ However, in their studies, they were only observing out-of-plane defects and no in-plane defects, likely due to the much larger compressive strain of −2.6% instead of −1.4%. In our cross-sectional samples, we also see dark stripes and they also always coincide with tail-to-tail CDWs (*e.g*., Figures 2, 3, and 4). Additionally, we see a very slight reduction in the O–K edge intensity of the EELS signal, which could be a direct hint of a reduced oxygen content (Figure 5c). These are apparent indications that the origin of the dark stripes in our samples is also the accumulation of oxygen vacancies.

Campanini *et al.* (see Figure 6b) also observed these dark stripes in their HAADF images in a Bi_0.8_Ca_0.2_FeO_3_ sample. Mapping of the A-site positions showed that across the dark stripes, A–A distances are alternating, having one larger elongation followed by a smaller one.^19^ Campanini *et al.* presented a model structure explaining the A–A distance oscillations with one O site in the FeO_2−δ_ plane having few vacancies and the next site having many vacancies, as schematically depicted in Figures 3d and 4e. The Fe atoms move further away from the site with many vacancies and increase the adjacent A–A distances.^19^ This model was received by directly mapping the O column intensity in ABF (annular bright field) STEM imaging.^19^ Mapping the A-site positions in our sample across the dark stripes, we also observe alternating A–A distances with one A–A pair showing a larger elongation and the subsequent one being smaller (Figures 3e and 4h). Thus, this alternating pattern is another evident indication that the nature of these defects is indeed the planar arrangement of oxygen vacancies. These columns with a high amount of oxygen vacancies form one-dimensional (1D) channels oriented in [010]_pc_ direction. Also, the same type of oxygen vacancy channels has been found in 50 at. % doped Bi_0.5_Ca_0.5_FeO_3_.^22^ It is important to note that while Brownmillerite-like defects have been observed in many perovskite materials, such as, for example, LaCoO_3−δ_-based,^61−64^ SrFeO_3−δ_-based,^65^ or LaMnO_3−δ_-based,^66^ the oxygen vacancy ordering, which would be the explanation for the structure observed in this study, does not belong to the Brownmillerite-like ordering because the O vacancy channels are oriented in the [010]_pc_ direction and not in the [110]_pc_ direction.^66^

Geng *et al.* (see Figure 6c) saw a lattice elongation in the dark stripes of around 11%, which they assigned through density functional theory calculations in the FeO_2−δ_ layers to a value of δ = 1.^32^ In our film, we also measured a lattice elongation for in-plane and out-of-plane defects of approximately 11%, which also hints on a value of δ ≈ 1. According to the result of Campanini *et al.*, the alternating A–A distances indicate that in the FeO_2−δ_ layers at the center of the dark lines in the HAADF image (*e.g.*, Figures 2, 3, and 4), every second oxygen site has many vacancies, while the other have few (schematic disposition in Figures 3d and 4e), suggesting also a value of δ ≈ 1. Therefore, based on the literature data, the alternating A–A distances as well as the 11% lattice elongation in our sample both in consistency suggest a value of δ ≈ 1.

The intensity analysis of the Fe sites within the defects (see Figure 2e and the blue curves in Figures 3b and 4b) shows that its values are reduced by 21 ± 10% compared to the undistorted structure, resulting in the darker contrast. Simulations of a model Bismuth ferrite structure with undistorted regions and defects with a larger A–A spacing but without Fe vacancies using the software Dr. Probe^46^ show an intensity decrease by 14%, which is within the variance of the measurement results (see Figure S4). Hence, Fe vacancies as the reason for the reduced Fe-site intensities within the defects can be discarded. Looking at the A-site intensities of the HAADF images, where Bi atoms and Ca dopants are sitting, their red curves in Figures 3b and 4b show a reduction of intensities around the defect. Opposite to the Fe-site intensities, the simulations show no change in the Bi-site intensities at the defect compared to those of the undistorted structure (see Figure S4). Consequently, these intensity reductions indicate either an agglomeration of Ca or an agglomeration of Bi vacancies at the A-sites adjacent to the defect, similar to the behavior observed by Campanini *et al.*(19) Because Ca is an earth alkali metal, it offers only two electrons in the sp valence complex and does not exceed the 2+ oxidation state. Hence, Ca sitting on the Bi^3+^ positions or Bi vacancies act as charge compensation for the oxygen vacancy rich defects, and can explain, why we see no mixed valence states of Fe^3+^ and Fe^2+^.^32,67^

The oxygen-deficient defect stripes in our 10 at. % Ca-doped Bi_0.9_Ca_0.1_FeO_3−δ_ thin film show a random spacing between each other and no periodicity, like it has been reported for higher Ca dopant ratios.^19,28^ While here the defects always coincide with CDW within the ferroelectric structure of the film, it does not seem to be the case for higher dopant ratios.^19^ Earlier studies on oxygen vacancies in epitaxially grown LaCoO_3−δ_ films suggest that under compressive strain from the substrate, out-of-plane oxygen defects appear and under tensile strain in-plane defects occur.^19,26^ Here, the oxygen-deficient defects appear simultaneously in both directions. We expected to see a difference and preference between in-plane and out-of-plane line defects either concerning the amount of oxygen vacancies δ or concerning their exact arrangement. However, the observations indicate no differences concerning these two characteristics. The fact that the ordered oxygen vacancy plates coincide with CDWs in the film could be useful to control domains and domain walls in multiferroics,^68^ due to the pinning effect of oxygen vacancies on domain walls.^36−38^ Because charged defects, of which oxygen vacancies are one type, have shown to have a critical influence on the electrical conductivity of domain walls if they are agglomerated in the domain wall region, our results are also relevant for the field of domain wall nanoelectronics.^69,70^

## 5 Conclusions

In conclusion, we have shown that a Bi_0.9_Ca_0.1_FeO_3−δ_ film under compressive strain shows oxygen vacancies agglomerated in plates with an alternating pattern concerning the [010]_pc_ oriented oxygen-site columns. Thereby, one oxygen column contains few vacancies and the two adjacent columns contain many vacancies forming vacancy channels. Even though the film is under compressive strain from the STO substrate, not just out-of-plane oxygen-deficient plates exist but also in-plane ones. Besides their different orientation, the ordered oxygen vacancies show the same ordering and oxygen vacancy concentration. The oxygen-deficient plates coincide with charged domain walls in tail-to-tail configuration. This leads to a pinning effect of the domain walls, causing critical aspects for device applications such as fatigue phenomena and countering of retention failure. This could be intentionally used to design properties in functional devices. The agglomeration of the oxygen vacancies at the domain wall could influence the domain wall conductivity and be relevant for domain wall nanoelectronics. Finally, our results give interesting insights into the functionalities, mechanisms, and interactions of charged domain walls and ordered oxygen vacancies.

## Supporting Information Available

STEM images and strain analysis about the epitaxial growth quality of the thin film system, versions of Figures 2, 3, and 4 optimized for colorblind readers, crystallographic data for the supercell model for the Dr. Probe^46^ HAADF image simulations, HAADF image simulation results, and two HAADF images to compare the BCFO film of this study with an undoped BFO film with comparable fabrication parameters (PDF)
