# Supplementary material for: Negatively Charged In-Plane and Out-Of-Plane Domain Walls with Oxygen-Vacancy Agglomerations in a Ca-Doped Bismuth-Ferrite Thin Film
Source: ACS Appl Electron Mater. 2021 Sep 24;3(10):4498–508. doi: 10.1021/acsaelm.1c00638 (PMC8552442; doi:10.1021/acsaelm.1c00638)
Supplement: Supplementary file 1 — el1c00638_si_001.pdf [file el1c00638_si_001.pdf]

Supporting Information

Negatively-Charged In-Plane and Out-of-Plane Domain Walls with Oxygen-Vacancy Agglomerations in a Ca-doped Bismuth-Ferrite Thin Film

Ulrich Haselmann[1], Y. Eren Suyolcu[2,3], Ping-Chun Wu[4], Yurii P. Ivanov[1,5,6], Daniel Knez[7], Peter A. van Aken[3], Ying-Hao Chu[4] and Zaoli Zhang[1,8]*

1 Erich Schmid Institute of Materials Science, Austrian Academy of Sciences, 8700 Leoben, Austria

2 Department of Materials Science and Engineering, Cornell University, Ithaca, New York 14850, United States

3 Max Planck Institute for Solid State Research, 70569 Stuttgart, Germany

4 Department of Materials Science and Engineering, National Chiao Tung University, Hsinchu 30010, Taiwan

5 Department of Materials Science & Metallurgy, University of Cambridge, Cambridge CB3 0FS, UK

6 School of Natural Sciences, Far Eastern Federal University, 690950, Vladivostok, Russia

7 Graz Centre for Electron Microscopy, Austrian Cooperative Research, 8010 Graz, Austria

8. Institute of Material Physics, Montanuniversität Leoben, 8700 Leoben, Austria

*Corresponding author: zaoli.zhang@oeaw.ac.at

In Table. S1 complementary imaging parameters of the HAADF data, which have not been mentioned in section 2. *Experimental Details* in the subsection *2.2. Data acquisition* are listed. For

Figure 3 five subsequent images each with 1.4 μs pixel time are summed up, resulting in a total pixel time of 7 μs.

Table. S1. *Complementary imaging parameters of the STEM HAADF images.*

	collection angle [mrad]	pixel time [μs]	step size [pm]	size [pixel*pixel]
Figure 1	75-309	10.2	58	1536 * 1024
Figure 2	83-205	10.2	12	1024 * 1536
Figure 3	75-309	5 * 1.4 = 7	7	1024 * 1024
Figure 4	83-205	13.6	7.2	1536 * 768
Figure 5	110-270	61	36	512 * 512
Figure S1	110-270	6.8	58	1536 * 1536
*Figure S5*a	75-309	6.8	58	1536 * 1536
*Figure S5*b	110-270	5.1	35	1536 * 2048

The High Angle Annular Dark Field (HAADF) image in Figure S1a shows on a lamella in $[110]_{pc}$ zone axis that the $SrRuO_3$ (SRO) interlayer and the $Bi_{0.9}Ca_{0.1}FeO_3$ (BCFO) layer have grown in a good epitaxial quality on the $SrTiO_3$ (STO) substrate. The blue dashed lines indicate the interfaces between STO and SRO and SRO and BCFO. GPA analysis for the in-plane strain in Figure S1b shows that there is only a slight in-plane relaxation from the beginning of the SRO layer to the first 20 nm of the BCFO. In comparison, the out-of-plane strain in Figure S1c shows a significant increase with the beginning of the SRO layer, and is only slightly reduced with larger film thickness. The scanning frame was once rotated by 90° so that each for ε_{xx} and ε_{zz} the primary scanning direction was normal to the direction of the analyzed strain. The Bragg vectors (1-10) and (002) were used, respectively.

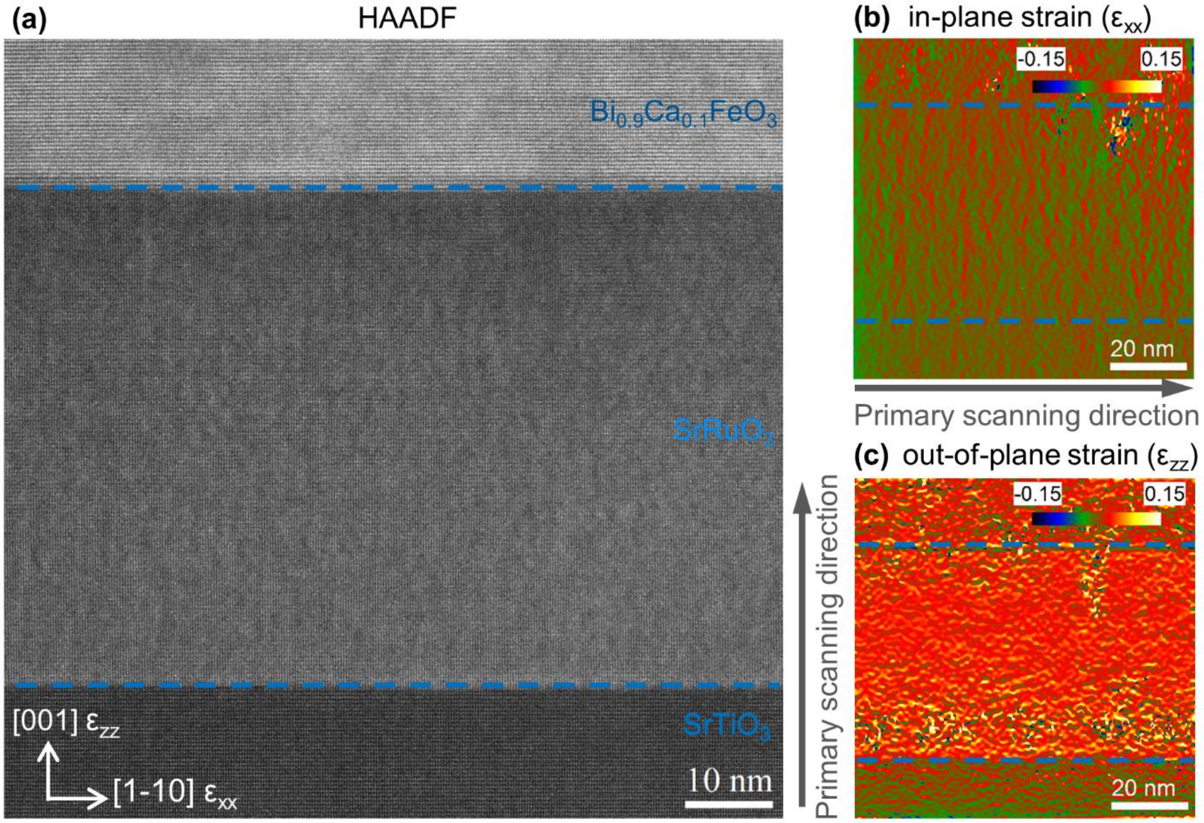

Figure S1. *Epitaxial growth quality of the thin film system. (a) HAADF image of a lamella from the pseudo-cubic [110]$_{pc}$ direction. (b) In-plane strain of the film system showing only slight increase along increasing layer thickness. (c) Out-of-plane strain, where clearly a significant increase at the STO-SRO interface can be seen with only slight decrease across the layer thickness. For the GPA analysis, the Bragg vectors (1-10) and (002) were used. The blue dashed lines indicate the respective interfaces.*

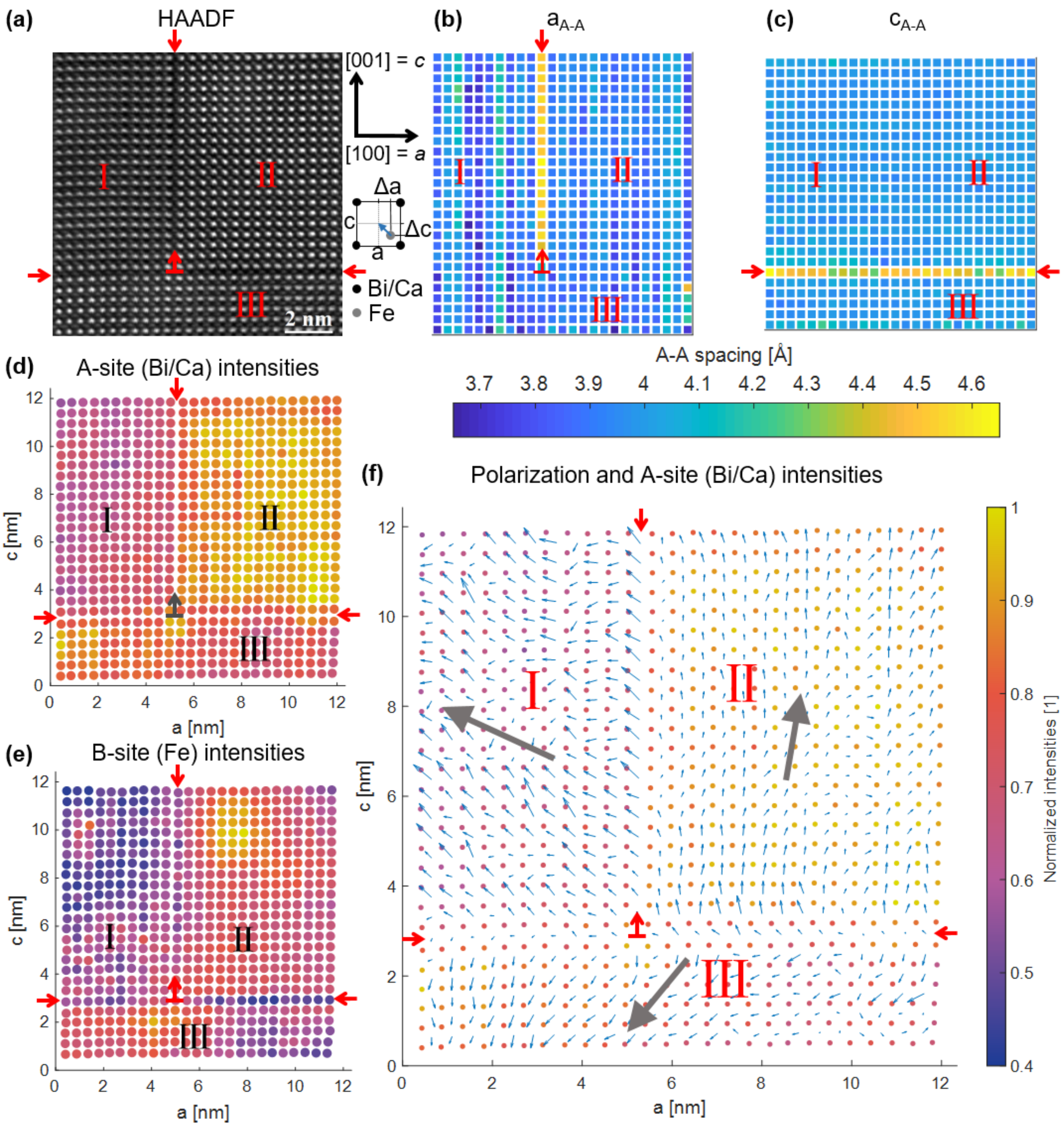

Figure S2 *Version of Figure 2 from the main manuscript changed in (d), (e) and (f) to optimize the display for colorblind readers. It shows the analysis of high-resolution STEM data of an in-plane and out-of-plane defect, which have a common point of contact (a) HAADF image showing two defects marked with red arrows. The defects separate the image into three areas designated with roman numbers I-III. On the top right side of the HAADF image, the $[100]_{pc}$ and $[001]_{pc}$ axes are indicated as a and c. At the bottom of the right side is a schematic illustration of the electrical polarization due to shift of the Fe atom from the center of the pseudo-cubic cell. (b) Map of*

interatomic distances of the A-sites (Bi and Ca atoms) in a (in-plane)-direction. The in-plane defect marked by the red arrows can be clearly seen by the enlarged lattice parameter. (c) Map of interatomic A-site distances in c (out-of-plane)-direction. The out-of-plane defect can be clearly observed by the enlarged lattice parameter. (d) A-site intensities (Bi and Ca positions) in the HAADF image normalized to the maximal A-site intensity. (e) B-site (Fe) intensities in the HAADF image normalized to the maximal B-site intensity. (f) Map of the electrical polarization due to the shift of the Fe atom overlayed on the fitted positions and intensities of the A-site atoms. The grey arrows in the regions I-III indicate the average polarization direction and magnitude relative to each other.

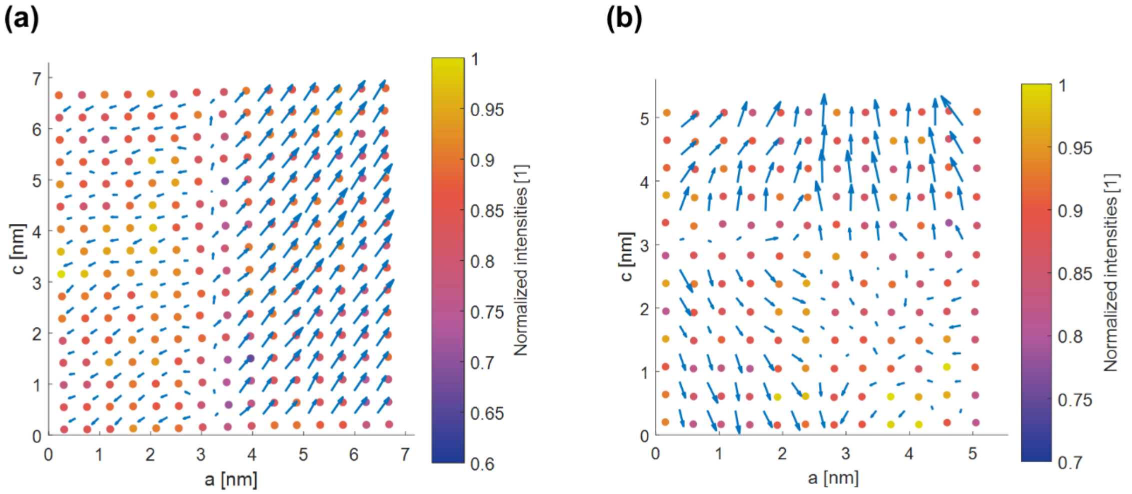

Figure S3 *Colorblind version of (a) Figure 3h and (b) Figure 4f.*

In Figure S4 the results of HAADF image simulations using the software Dr. Probe[1] are shown. The cation positions of the crystallographic supercell model used for the simulations is depicted in Figure S4a. It was created by multiplying the pseudo-cubic BFO unit cell three times in the c direction and modifying the Bi and Fe positions to replicate the defect, its lattice elongation with a value of 12.5% compared to the undistorted lattice and a changed polarization state (polarization within the defect was assumed zero). The thereby modified supercell became in the strict crystallographic sense a new modified unit cell, but we continue to call it small supercell in the text. Its crystallographic data and atomic positions can be found in Table S2. This small supercell

(indicated by the grey rectangle in Figure S4a) was then multiplied 2 times each in the a and c directions to create the supercell model for the image simulations.

The result of the image simulations for a sample thickness of ≈ 30 nm (76 BFO unit cells), an acceleration voltage of $U_{acc} = 200$ kV, a convergence angle of $\alpha = 20.9$ mrad, a spherical aberration of $C_S = 0$ µm, a defocus value of 0 nm, an electron source radius of $r_s = 0.010$ nm, a scanning step size of 10 pm and a HAADF detector ranging from 49.0 - 250.0 mrad, can be seen in Figure S4b. The pairs of orange arrows in Figure S4a and Figure S4b indicate the area of the defect, where the lattice is elongated and the Fe is not shifted from the center meaning no local polarization there. A comparison of the intensities of the Fe sites in Figure S4c (area of the intensity profile is marked by the magenta rectangle in Figure S4b) shows that at the position in the defect (marked by the red arrow) it is significantly reduced compared to the iron intensities in the undistorted structure. This intensity reduction happens without a change of atomic composition within this column. For the ideal incoherent scattering case, the detected intensity would be proportional to $Z_1^2 + Z_2^2 + \ldots + Z_n^2$ with Z_k being the atomic number of each atom in the column and would not be influenced by an enlarged lattice spacing. In contrast, Figure S4d shows that the intensities of the Bi sites are not influenced by the defect (the area of the intensity profile is marked by the turquoise rectangle in Figure S4b). This is important to keep in mind, since a change in the Bi site intensities of the recorded images means, opposite to the Fe intensities, a compositional change.

Table S2. *Crystallographic data of the small supercell created by multiplying the pseudocubic BFO unit cell 3 times in c direction (marked by the grey rectangle in Figure S4a). The cell parameters are a = b = 3.96 Å, c = 11.88 Å, α = β = γ =90°.*

Atom site label	Atom site type symbol	Occupancy	Atom site fraction x	Atom site fraction y	Atom site fraction z
Bi1	Bi	1	0	0	0
Bi2	Bi	1	0	0	0.32
Bi3	Bi	1	0	0	0.64
Fe1	Fe	1	0.65	0.65	0.208
Fe2	Fe	1	0.65	0.65	0.528
Fe3	Fe	1	0.5	0.5	0.82
O1	O	1	0	0.5	0.16
O2	O	1	0.5	0	0.16
O3	O	1	0.5	0.5	0
O4	O	1	0.5	0.5	0.32
O5	O	1	0	0.5	0.48
O6	O	1	0.5	0	0.48
O7	O	1	0.5	0.5	0.64
O8	O	1	0	0.5	0.82
O9	O	1	0.5	0	0.82

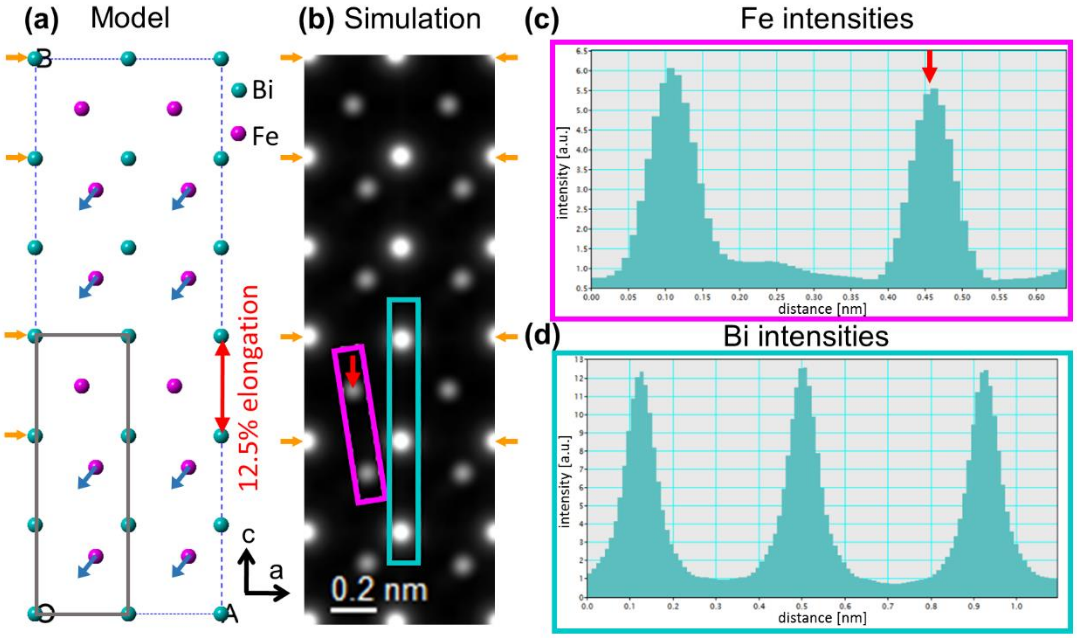

Figure S4. *STEM image simulation with the software Dr. Probe*[1] *(a) Cation model of the supercell used for the simulations (in this depiction the oxygen atoms are faded out for more clarity). The grey rectangle indicates the small supercell, which is multiplied with two in the a and c directions to receive the big supercell covering the whole simulation area. The small supercell consists of 3 pseudo-cubic BFO unit cells, where the atom positions have been modified to model the defect (indicated by the 2 pairs of orange arrows on the left side) and the undistorted cells. The arrows indicate a shift of the central Fe atom and therefore a local polarization. (b) HAADF (49-250 mrad) simulation result from Dr. Probe*[1] *for a sample thickness of 30 nm and a step size of 10 pm. (c) HAADF intensity of the Fe atoms in the area of the magenta rectangle in (b), which is significantly reduced for the Fe atom in the defect area marked by the red arrow. (d) HAADF intensity of the Bi atoms from the turquoise rectangle in (b), which show no influence from the defect.*

Figure S5 shows an overview HAADF image of the BCFO film from this study in comparison with an overview HAADF image from an undoped BFO reference sample. The BFO reference sample was deposited on the same SRO-STO substrate system with the same deposition parameters as the BCFO film with the exception of 15 min deposition time (instead of 45 min)

resulting with 20 nm in a third of the thickness. While the BCFO sample clearly shows agglomerated oxygen vacancies, the undoped BFO film shows no agglomerated oxygen vacancies.

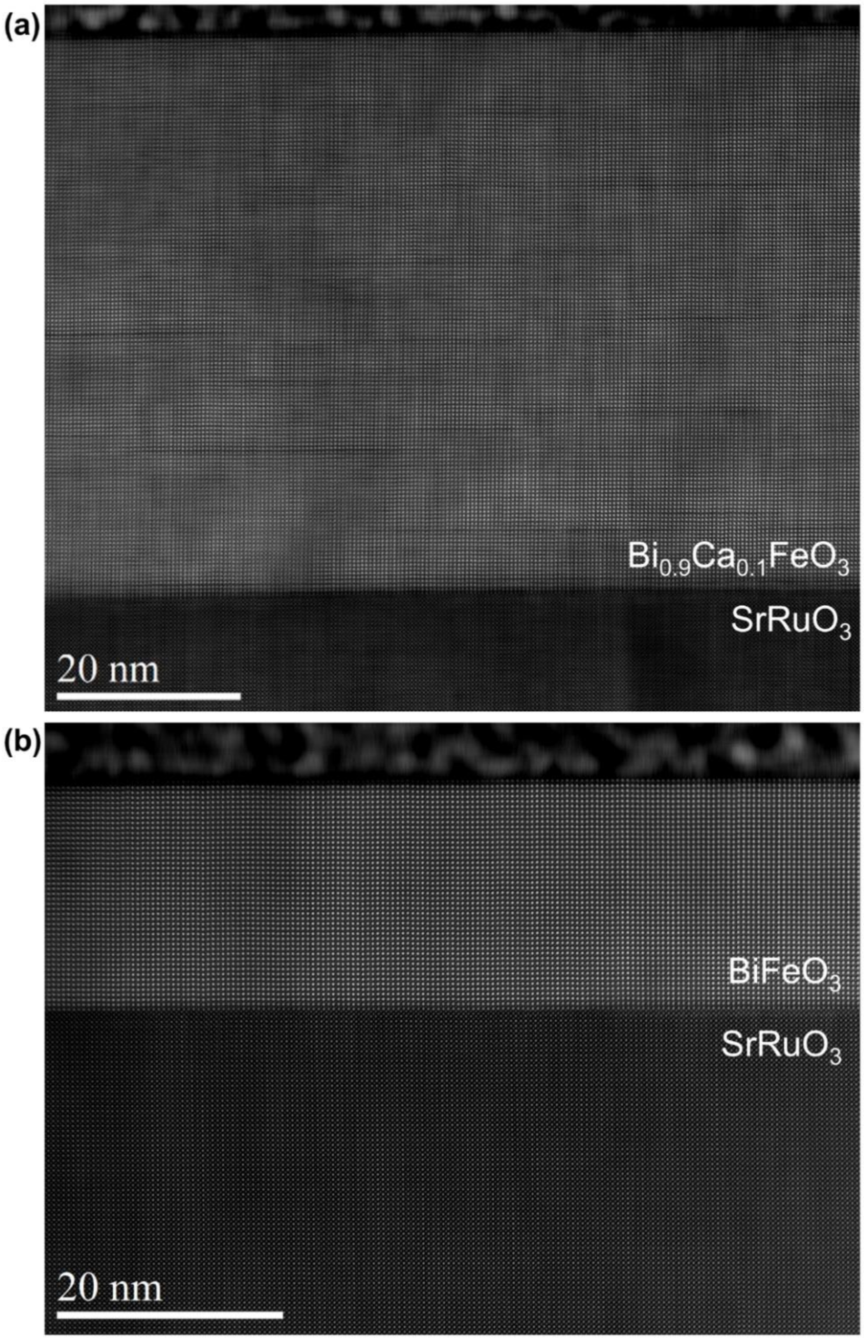

Figure S5. *HAADF image comparison of (a) the BCFO film of this study with (b) an undoped BFO film on the same SRO-STO substrate. While the Ca doped film shows agglomerations of oxygen vacancies throughout the film, the BFO film shows none.*

References

(1) Barthel, J. Dr. Probe: A Software for High-Resolution STEM Image Simulation. *Ultramicroscopy* **2018**, *193*, 1–11.
